# Low Serum Glutathione Peroxidase Activity Is Associated with Increased Cardiovascular Mortality in Individuals with Low HDLc’s

**DOI:** 10.1371/journal.pone.0038901

**Published:** 2012-06-15

**Authors:** Brian Buijsse, Duk-Hee Lee, Lyn Steffen, Richard R. Erickson, Russell V. Luepker, David R. Jacobs, Jordan L. Holtzman

**Affiliations:** 1 Division of Epidemiology and Community Health, School of Public Health, University of Minnesota, Minneapolis, Minnesota, United States of America; 2 Department of Epidemiology, German Institute of Human Nutrition Potsdam-Rehbruecke, Nuthetal, Germany; 3 Department of Pharmacology, University of Minnesota, Minneapolis, Minnesota, United States of America; 4 Department of Medicine, University of Minnesota, Minneapolis, Minnesota, United States of America; 5 Division of Environmental Health Sciences, University of Minnesota, Minneapolis, Minnesota, United States of America; 6 Division of Preventive Medicine, School of Medicine, Kyungpook National University, Jung-gu, Daegu, South Korea; 7 Department of Nutrition, University of Oslo, Oslo, Norway; University of Freiburg, Germany

## Abstract

**Background:**

Since oxidized LDL is thought to initiate atherosclerosis and the serum glutathione peroxidase (GPx3) reduces oxidized lipids, we investigated whether high GPx3 activity reduces cardiovascular disease (CVD) mortality.

**Methods:**

We determined GPx3 in stored samples from the Minnesota Heart Survey of 130 participants who after 5 to 12 years of follow-up had died of CVD and 240 controls. Participants were 26 to 85 years old and predominantly white. In a nested case-control, study we performed logistic regressions to calculate odds ratios (OR) adjusted for age, sex, baseline year, body mass index, smoking, alcohol intake, physical activity, total and HDL cholesterols, systolic blood pressure, serum glucose and gamma glutamyltransferase (GTT) activity. The referent was the quartile with the highest GPx3 activity (quartile 4).

**Results:**

OR’s for CVD mortality for increasing quartiles of GPx3 were 2.37, 2.14, 1.83 and 1.00 (P for trend 0.02). This inverse correlation was confined to those with HDLc’s below the median (P for interaction, 0.006). The OR’s for increasing quartiles of GPx3 in this group were 6.08, 5.00, 3.64 and 1.00 (P for trend, 0.002).

**Conclusions:**

Individuals with both low HDLc and GPx3 activity are at markedly increased risk for death from CVD.

## Introduction

It is widely held that oxidative stress is a major factor in the vascular injury that leads to atherosclerosis and CVD [1] In spite of the failure of dietary antioxidants to decrease CVD incidence [2], there is still a significant body of evidence suggesting that oxidative injury has a major role in the initiation of atherosclerosis [3]. These findings include the observation that oxidized serum lipid levels are higher in patients with CVD than in controls [3], [4]–[6]. Similarly, Salonen *et al*. [7] have reported that patients have antibodies to oxidized lipids suggesting that they have a greater exposure to these toxic products than do controls. If oxidized lipoproteins are a major factor in the development of atherosclerosis, then endogenous systems that reduce the levels of these toxic products may be important in the prevention of CVD.

The glutathione peroxidases (GPx) are a family of enzymes which reduce oxidized lipids to their nontoxic metabolites and may thereby decrease vascular injury [3]. The two major forms are GPx1 and GPx3. GPx1 is found solely in the cytosol and mitochondria, whereas GPx3 is only present in HDL particles [3], [8]. They reduce oxidative stress by catalyzing the glutathione dependent reduction of lipid hydroperoxides to their corresponding alcohols and hydrogen peroxide to water [3].

Previous studies have suggested that low levels of both GPx1 and GPx3 are associated with the development of vascular disease. For example, in the Athero Gene study of patients with a history of CVD, those with low erythrocyte GPx1 activities had an increased incident of recurrent events [9], [10]. Similarly, in a case-control study, Voetsch *et al*. [11] found that several single nucleotide polymorphisms (SNP’s) in the GPx3 gene promoter region, which decreased the expression of this enzyme [12], were associated with an increased incidence of ischemic stroke before the age of 45. Yet, no prospective, population based studies on the association between GPx3 activity and CVD have been reported.

We have performed a nested, case-control analysis of stored serum samples from the Minnesota Heart Survey to determine whether high GPx3 activity may reduce CVD mortality. Consistent with its presumed role in reducing oxidative injury, we hypothesized that a higher activity would be associated with a lower incidence of CVD. In a subgroup analysis, we anticipated that there may be a greater inverse correlation between outcomes in those with low and high HDLc’s, since those with low levels are likely to need additional antioxidant activity in order to prevent CVD.

**Table 1:** Baseline characteristics of the study population by case-control status, in the Minnesota Heart Survey.*

Characteristic	Cases (N = 130)	Controls (N = 240)	P†
Males – % (no.)	57.7 (75)	56.7 (136)	0.85
Age – yr	69.2±11.9	67.9±12.2	0.33
BMI – kg/m^2^	27.8±5.1	27.3±4.3	0.16
Current smoker – % (no.)	20.8 (27)	8.3 (20)	0.0006
Alcohol – drinks per week	5.6±12.8	3.4±6.8	0.07
Physical activity – MET-hr/day	8.5±12.7	7.8±10.7	0.60
Serum cholesterol – mg/dl			
Total	220±43	216±38	0.43
HDLc	41±14	45±15	0.03
Lipid-lowering medication – % (no.)	13.8 (18)	6.7 (16)	0.02
Hypercholesterolemia – % (no.)∥	41.5 (54)	32.1 (77)	0.06
Blood pressure – mmHg			
Systolic	135.9±18.5	130.1±18.9	0.005
Diastolic	75.7±12.7	74.6±10.6	0.37
Antihypertensive use – % (no.)	40.8 (53)	25.4 (61)	0.002
Hypertension - % (no.)§	66.9 (87)	43.8 (105)	<0.0001
Self-reported diabetes – % (no.)	19.2 (25)	7.5 (18)	0.0008
Aspirin use – % (no.)	39.2 (51)	35.0 (84)	0.52
Enyme activities in serum			
GPx3– U/ml, mean ± SE¶	0.34±0.01	0.37±0.01	0.02
GGT – U/l	43.3±69.7	25.9±18.3	0.006
Non-fasting serum glucose – mg/dl	130±62	109±36	0.0008
Urea nitrogen – mg/dl	19.7±9.2	18.1±4.9	0.07

* Value are the means±SD, unless otherwise indicated.

† Based on 2-sample t-test, Mann-Whitney U test, or Chi-square test.

§ Defined as a blood pressure of ≥140/90 mm Hg or the use of antihypertensive medication.

∥ Defined as a serum total cholesterol >200 mg/dl.

¶ Means for the activity of GPx3 (standard error) were adjusted for baseline year in a covariance analysis.

**Table 2:** Cross-sectional relation between GPx3 activity and characteristics at baseline: the Minnesota Heart Survey.*

Characteristic	Baseline Quartiles of serum GPx3 activity †				P trend ‡
	Q1	Q2	Q3	Q4	
No. of controls	60	61	60	59	
Males – % (no.)	60.0 (36)	55.7 (34)	61.7 (37)	49.2 (29)	0.81
Age – yr	66.6±11.3	68.4±13.5	68.1±12.8	68.5±11.1	0.52
BMI – kg/m^2^	27.7±4.4	26.8±4.6	26.9±3.4	27.8±4.8	0.46
Current smoker – % (no.)	8.3 (5)	6.6 (4)	11.7 (7)	8.5 (5)	0.86
Alcohol – drinks per week	2.5±6.1	3.2±5.1	4.7±9.4	3.1±5.6	0.33
Physical activity – MET-hr/day	9.1±12.2	7.0±9.8	6.8±9.5	8.2±11.4	0.63
Blood pressure – mmHg					
Systolic	133.9±20.7	130.6±19.4	128.2±16.9	127.8±18.4	0.26
Diastolic	75.3±10.0	75.4±11.6	74.0±9.6	73.5±11.3	0.71
Antihypertensive use – % (no.)	30.0 (18)	24.6 (15)	20.0 (12)	27.1 (16)	0.64
Hypertension - % (no.)§	51.7 (31)	50.8 (31)	33.3 (20)	39.0 (23)	0.11
Serum cholesterol – mg/dl					
Total	214±36	219±34	213±43	219±39	0.73
HDLc	43±14	50±14	45±14	42±15	0.03
Lipid-lowering medication – % (no.)	10.0 (6)	0.0 (0)	11.7 (7)	5.1 (3)	0.04
Hypercholesterolemia – % (no.)∥	28.3 (17)	31.1 (19)	35.0 (21)	33.9 (20)	0.89
Self-reported diabetes – % (no.)	10.0 (6)	4.9 (3)	6.7 (4)	8.5 (5)	0.74
Aspirin use – % (no.)	33.3 (20)	36.1 (22)	23.3 (14)	42.4 (25)	0.17
GGT – U/l	28.1±21.6	26.2±17.2	23.6±14.0	25.7±19.8	0.61
Non-fasting serum glucose – mg/dl	115±55	110±30	105±24	108±26	0.47
Urea nitrogen – mg/dl	17.4±3.9	17.7±4.4	17.8±3.8	19.6±6.8	0.05

* The values are the means±SD, unless otherwise indicated.

† Shown are the year-specific quartiles of GPx3 activity, based on the distribution in controls. Ranges of GPx3 activity in U/ml for increasing quartiles were 0.09–0.23, 0.24–0.30, 0.31–0.42, and 0.43–0.76 in 1990–1992, and 0.17–0.35, 0.36–0.47, 0.48–0.56, and 0.57–1.03 in 1995–1997.

‡ Based on modeling quartiles of GPx3 activity as a continuous variable.

§ Defined as a blood pressure of ≥140/90 mm Hg or the use of antihypertensive medication.

∥ Defined as a serum total cholesterol >200 mg/dl.

## Methods

### Subject Selection

The subjects were participants in the Minnesota Heart Survey. Methods of this study have been previously published [13]–[15]. Briefly, it was initiated in 1980 as an ongoing population-based surveillance survey on the trends of risk factors for CVD in residents of the seven counties of the Minneapolis-St. Paul metropolitan area. A two-stage, self-weighting cluster design was used to randomly select households. Within each household, one individual was randomly selected to participate in the survey except for the 1980–1981, 1995–1997, and 2000–2002 surveys in which all age-eligible household members were invited to participate [16]. Since the subjects were not seen after their initial evaluation, the follow-up mortality was determined by matching subjects with the Minnesota death certificates filed on or before December 31, 2002. Written consents were obtained from all study participants. The consents and data collection procedures for each survey were approved by the University of Minnesota Research Subjects’ Protection Programs Institutional Review Board.

For this study, we used data and stored serum samples from the 1990–1992 and 1995–1997 surveys. We identified 173 CVD deaths (International Classification of Diseases ninth revision codes 390–459 or tenth revision codes I00–I99) and two age and sex matched controls per case (n = 346). Serums were available from 137 of the participants who had died and 250 controls. Three controls died during follow-up from causes other than CVD. The subjects ranged in age from 26 to 85 years. Missing blood samples occurred more frequently in the 1995–1997 survey than in the earlier collection period. There were 96 cases and 198 controls from the 1990–1992 survey and 41 cases and 51 controls from the 1995–1997 survey. Complete covariate data were available in 130 cases (95 in 1990–1992 and 35 in 1995–1997) and 240 controls (197 in 1990–1992 and 43 in 1995–1997). Findings were essentially unaffected if the 16 missing covariate values were imputed from the participants’ sex, age, body mass index (BMI), and diabetes status, but we report our findings only from those with a complete data set because 12 of the missing values were for their HDLc levels, a variable which we had postulated could play a role in our analysis.

### Clinical Assessment

Subjects’ heights were measured in their stocking feet with a wooden triangle by a rigid ruler attached to the wall. Weight was measured with a beam balance without the subjects wearing either a coat or shoes. The BMI was calculated as the weight in kg divided by the square of the height in m.

Systolic and fifth phase diastolic blood pressures were measured with a random zero sphygmomanometer (Hawksley, West Sussex, United Kingdom) by trained technicians according to a standard procedure [16]. The recorded blood pressures were the average of two measurements taken 1 minute apart.

Information on age, sex, leisure-time physical activity, smoking, and alcohol consumption was obtained by interviewer-administered questionnaires. For leisure-time physical activity, questions were asked about the intensity, duration, and frequency of exercise. Using these data we derived a physical activity score in MET-hr/day [17].

### Laboratory Serum Assays

Blood was drawn from non-fasting subjects according to standardized protocols that differed in one important respect between the 1990–1992 and 1995–1997 surveys. In the 1990–1992 survey serum samples were refrigerated at 4°C for up to several days before freezing while in the 1995–1997 survey they were frozen at −70°C within 24 hours. Upon receipt in the laboratory, all serum samples were stored at −70°C. Serum was used for all laboratory analyses.

Total serum cholesterol was determined with an AutoAnalyzer II (Technicon Corporation) by a nonenzymatic method in 1990–1992 and an enzymatic method thereafter. HDLc was determined by an enzymatic method after heparin and Mn2+ (1990–1992) [18] or magnesium dextran sulfate (1995–1997) precipitation of non-HDLc [19]. Glucose, urea nitrogen, aspartate aminotransferase (AST), alanine aminotransferase (ALT) and GGT were determined in a Vitros 950 multi-channel analyzer (Ortho Clinical Diagnostics, Raritan, NJ) by the manufacturer’s standard thin-layer reflectance spectrophotometric methods. Average analytical coefficient of variation (CV) for these assays at the high end of the normal range were less than 3%.

GPx3 activity was determined by a modified kinetic assay as previously described [8]. Briefly, serum samples were aliquoted in quadruplicate into 96 well microtiter plates. Glutathione and glutathione reductase were added. After warming to 37°C NADPH and *t*-butyl hydroperoxide, were added. The decrease in absorbance at 340 nm was determined kinetically in a microtiter plate reader (FLUOstar, BMG, Offenberg, Germany). The CV’s were 5% within each plate. We presumed that we were determining only the GPx3 activity since previous studies have reported that it was the only GPx in the serum [20], [21]. GPx3 activity was found to be lower in the 1990–1992 samples than those from 1995–1997 (see Results). The assay was consistent, in that the same survey differences occurred on the single assay plate that contained samples from both surveys (mean (SD) GPx3 activity 0.36 (0.13) U/ml in 13 samples from 1990–1992 compared with 0.76 (0.07) U/ml in eight samples from 1995–1997).

**Table 3:** Odds ratios (ORs) and 95% confidence intervals (CIs) of cardiovascular mortality by serum GPx 3 activity.

	Baseline Quartiles of serum GPx-3 activity *					
	Q1	Q2	Q3	Q4	Regression coefficient (SE) ‡	P
*All subjects*						
No. of cases/controls	43/59	35/60	32/61	20/60		
Odds ratio (95% CI)						
Model 1§	2.30 (1.20–4.94)	1.81 (0.93–3.50)	1.60 (0.82–3.11)	Referent	−0.30 (0.14)	0.03
Model 2∥	2.26 (1.16–4.43)	1.78 (0.90–3.54)	1.59 (0.79–3.18)	Referent	−0.28 (0.14)	0.05
Model 3¶	2.37 (1.16–4.86)	2.14 (1.03–4.42)	1.83 (0.87–3.84)	Referent	−0.30 (0.15)	0.04
						
*Low HDLcl* **						
No. of cases/controls	35/35	20/26	15/20	9/33		
Odds ratio (95% CI)						
Model 1§	3.79 (1.57–9.14)	2.80 (1.08–7.22)	2.73 (1.00–7.43)	Referent	−0.52 (0.19)	0.005
Model 2∥	4.33 (1.72–10.9)	3.39 (1.25–9.19)	3.05 (1.06–8.79)	Referent	−0.57 (0.20)	0.004
Model 3¶	6.08 (2.12–17.4)	5.00 (1.63–15.3)	3.64 (1.10–12.0)	Referent	−0.68 (0.22)	0.002
						
*High HDLc* **						
No. of cases/controls	8/24	15/34	17/41	11/27		
Odds ratio (95% CI)						
Model 1§	0.87 (0.29–2.56)	1.11 (0.43–2.86)	0.99 (0.39–2.50)	Referent	0.09 (0.22)	0.67
Model 2∥	0.78 (0.24–2.56)	1.02 (0.36–2.94)	1.04 (0.37–2.89)	Referent	0.15 (0.23)	0.53
Model 3¶	0.82 (0.23–2.89)	1.13 (0.38–3.36)	1.10 (0.39–3.13)	Referent	0.12 (0.24)	0.62

* Shown are the year-specific quartiles of GPx3 activity, based on the distribution in controls. Ranges of GPx3 activity in U/ml for increasing quartiles were 0.09–0.23, 0.24–0.30, 0.31–0.42, and 0.43–0.76 in 1990–1992, and 0.17–0.35, 0.36–0.47, 0.48–0.56, and 0.57–1.03 in 1995–1997. The highest quartile of GPx3 activity (quartile 4) was used as the referent.

‡ Shown are regression coefficients (standard errors) predicting the case-control logit per standard-deviation (0.16 U/ml) increase of GPx3 activity.

§ Adjusted for matching factors age (continuous) and sex, and baseline year.

∥ Adjusted as in model 1, but with additional adjustment for body mass index (continuous), current cigarette smoking, alcohol use (dummy variables in drinks per week: 1–6, 7–14, >14), and physical activity (continuous).

¶ Adjusted as in model 2, but with additional adjustment for total cholesterol (continuous), HDLc (continuous), systolic blood pressure (continuous), non-fasting glucose (continuous), and GGT (continuous). In stratified analysis, adjustment for HDLc was omitted.

** Low HDLc was defined as lower than the median serum value of 38 mg/dl for men and 48 mg/dl for women, high serum HDLc as greater or equal to 38 mg/dl in men and 48 mg/dl in women.

### Data Analysis

Case-control differences in GPx3 activity were similar within participants in the 1990–1992 and the 1995–1997 surveys, hence the primary findings were based on analyses of pooled data from the two surveys. However, given the difference in GPx3 activity between the two and the differences in the number of case-control blood samples that were available between the two collection periods, we also present survey specific data to demonstrate that our findings were consistent between surveys. In the pooled analyses, we adjusted for survey period and used baseline year-specific quartiles based on the distribution in controls in assessing the association between GPx3 activity and CVD mortality. Since our initial hypothesis was that high levels of GPx3 are protective, we elected to take the highest quartile as the referent. We report the odds ratios (OR) for CVD mortality calculated by logistic regression models. All analyses were adjusted for age, sex, and baseline year (model 1). In multivariable analyses, we first included the BMI, cigarette smoking, physical activity, and alcohol intake (model 2). This model was then extended by adding the values for the total HDLc’s, systolic blood pressure, non-fasting serum glucose levels and GGT activities (model 3). Trends across quartiles of GPx3 activity were assessed by modeling the GPx3 activity as a continuous variable. Stratified analyses for the HDLc were conducted to determine whether the association between GPx3 activity and CVD mortality differed between those above and below the sex-specific median values for the HDLc’s. This stratification was based on the hypothesis that both HDL and GPx3 may prevent vascular injury by removing oxidized lipids from the serum. If such is the case, then they could serve as complimentary antioxidant systems. The sex-specific median values for the HDLc were similar for the 1990–1992 and 1995–1997 surveys. A product term of GPx3 (quartiles) and HDLc (continuous) was entered into the multivariable model to assess the statistical significance of this interaction. All analyses were conducted with the SAS version 9.1 (SAS Institute Inc., Cary, NC).

**Figure 1:**
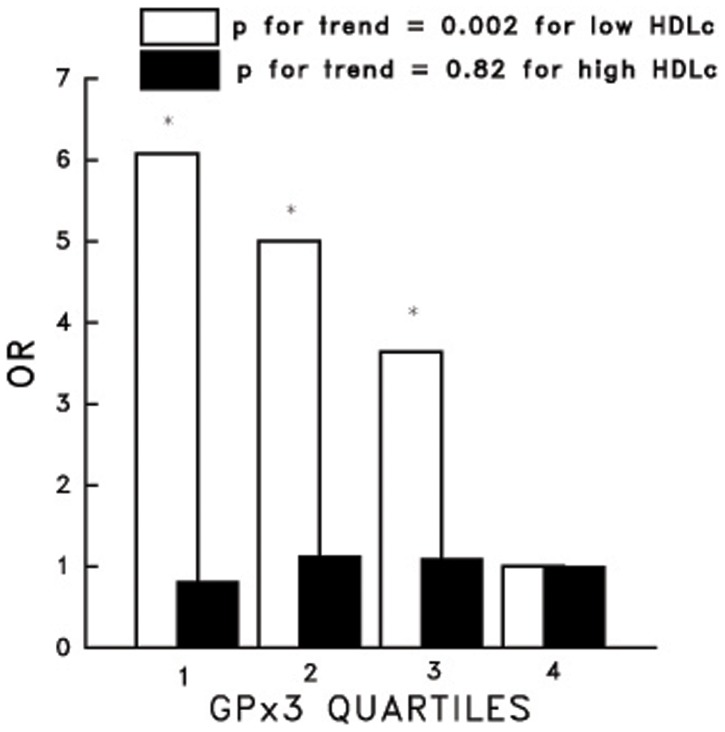
The Inverse Correlation between the GPx3 Activity by Quartile and the OR for Cardiovascular Disease Mortality in Individuals with High HDLc and Low HDLc. The trend in the odds ratios for CVD mortality versus the GPx3 activity as estimated by model 3 (see Methods) from the lowest quartile of GPx3 activity (quartile 1) to the highest (quartile 4) for individuals with sex specific HDLc at or above the median HDLc (38 mg% for males; 48 mg% for females) and below the sex specific HDLc. The referent is quartile 4. * P<0.05 as compared to the reference.

## Results

### Baseline Characteristics

Of the 130 CVD deaths included in our analyses, 66 (51%) had died of coronary heart disease, 25 (19%) of atherosclerotic stroke, 22 (17%) of other atherosclerotic CVD and 17 (13%) of non-atherosclerotic CVD. 95% of the cases and 97% of the controls were of white race. Cases were more likely to have smoked, had higher average systolic blood pressures, non-fasting serum glucoses and serum GGT’s [22] and lower average HDLc’s than did the controls (Table 1). Furthermore, they were more likely to have used medications for hypertension and hyperlipidemia, and self-reported having diabetes mellitus. These differences were mutually independent, apart from a higher prevalence of diabetes in the cases, which lost statistical significance after regression adjustment for the other risk factors for CVD. The GPx3 activities were lower in the cases than in the controls, but varied between surveys. The mean (SD) activity for samples collected in 1990–1992 was 0.32 (0.12) U/ml, while those collected in 1995–1997 had a mean value of 0.51 (0.19) U/ml. The case-control differences were generally similar between the two surveys. The AST and ALT activities were also higher in both cases and controls in the samples from 1995–1997 than those from 1990–1992. The survey differences for these three activities were most likely due to the differences in the handling of the serum samples. The GGT activities and serum lipids, glucose, and urea nitrogen concentrations were comparable between the two surveys (data not shown). Among controls, the GPx3 activity was unrelated or inconsistently related to the covariates studied (Table 2) and there were no notable differences in GPx3 associations with covariates between the 1990–1992 and 1995–1997 surveys (data not shown).

### Serum GPx3 Activity and Cardiovascular Mortality

The GPx3 activities were inversely and dose-dependently correlated with CVD mortality after controlling for the matching factors of age, sex, and baseline year (Table 3). Additional adjustment for conventional cardiovascular risk factors and GGT activity had little effect on the strength of this association. The regression coefficients predicting the case-control logit per standard deviation of GPx3 were consistently negative between the two survey periods: −0.48 (p = 0.02) in 1990–1992 and −0.10 (p = 0.70) in 1995–1997.

In a subgroup analysis, the association between GPx3 and CVD mortality was restricted to participants with below sex-specific median HDLc (cut points were 38 mg/dl in men and 48 mg/dl in women). There was no correlation between the GPx3 activity and CVD mortality in participants with higher HDLc’s (P for interaction, 0.006). For those with low HDLc’s, the multivariable adjusted regression coefficients predicting the case-control logit per standard deviation of GPx3 were consistently negative between survey years: −0.67 (p = 0.01) in 1990–1992 and −0.62 (p = 0.10) in 1995–1997, compared to the values for those at or above the median HDLc’s (−0.08 in 1990–1992 and 0.43 in 1995–1997.) Neither of these values was significantly different from zero.

When we examined the joint association of GPx3 activity and HDLc with CVD mortality, we took the highest GPx3 quartile (quartile 4) and the below median HDLc concentration as the single referent. Compared with this referent, the multivariable-adjusted risk for CVD mortality increased as the GPx3 activities decreased (Figure 1). Compared to those in the highest GPx3 quartile, the OR’s for the first, second, and third quartiles in this group were 6.08, 5.00, and 3.64, respectively (P for trend, 0.002). In contrast, in those at or above the median HDLc concentration, the risk for CVD mortality across quartiles of GPx3 was constant and similar to the risk seen in those with low HDLc and high GPx3 activity.

After excluding cases who had died of non-atherosclerotic CVDs (n = 13), the association between GPx3 activity and cardiovascular mortality in those with below median HDLc’s remained essentially the same compared to those in the lowest GPx3 quartile. The multivariable-adjusted OR’s in the first, second, and third quartile were 5.13, 4.23, and 2.96, respectively (P for trend, 0.003). Additional adjustments for the use of aspirin, medication for hyperlipidemia or hypertension, or the presence of diabetes mellitus did not affect these estimates. The association between GPx3 activity and CVD mortality was also not significantly different between smokers and non-smokers (P for interaction, 0.52), or when corrected for alcohol intake (P for interaction, 0.08) or age (P for interaction, 0.14).

## Discussion

In this nested, case-control, prospective study, the serum GPx3 activity was inversely and linearly correlated with CVD mortality, including coronary heart disease, other atherosclerotic disease and stroke. A subgroup analysis indicated that this association between low GPx3 activity and CVD mortality was only seen in subjects with low HDLc and was independent of conventional risk factors for CVD. We feel that these observations are consistent with the currently accepted role of oxidative injury in the development of atherosclerosis.

It is generally thought that oxidized plasma lipids initiate and promote atherosclerosis [1]. It would therefore be expected that any system, such as the GPx’s, which lower oxidized lipid levels should be protective. Hence, it is not surprising that a high serum GPx3 activity appears to prevent CVD mortality since it is the only GPx found in the serum [20], [21]. Although no previous population based studies have been published on the role of GPx3 in the prevention of CVD, a few studies have suggested that it may have a protective role [23]. For example, one study found that homocysteine decreased the concentration of nitric oxide through inhibition of GPx1 [24]. Similarly, Porter *et al*. [25] and Dogru-Abbasoglu *et al*. [26] reported that in two small cross-sectional studies, patients with CVD had lower plasma, platelet, and erythrocyte GPx activities than did controls. Since these were not prospective studies, there may have been confounding factors leading to their findings, including that the GPx3 activities may have declined as a result of the disease process rather than being its cause. Our study circumvented this problem, since our samples were collected before the subjects had known CVD.

Others have also highlighted the importance of glutatiione pathways in the prevention of vascular injury. For example, Voetsch *et al*. [11] reported that the presence of several SNP’s in the GPx3 gene promoter region which decreased its activity, were associated with an increased risk for premature ischemic strokes [12]. Finally, in a case-control study Morrison *et al*. [27] found that the serum glutathiione concentrations in the adolescent sons of men with a history of a recent myocardial infarction were significantly lower than those observed in the serum of sons of age-matched controls.

In subjects with low HDLc we found that a low GPx3 activity was associated with OR’s which would suggest that this combination may be a major risk factor for CVD. In fact those with this combination appear to be at a greater risk for CVD mortality than that attributed to moderate hypertension, diabetes mellitus, smoking a pack of cigarettes per day or a LDL in the 200 mg/dL range, where the OR’s are usually considered to be around 2–4 [28].

Our study has a number of major limitations. The most important is that the subjects were not seen in follow up after their initial visit. Hence we are unable to determine what effect the low HDL and GPx3 activity may have had on nonfatal CVD events. Similarly, we had no follow up on other possible effects of this combination, such as the intercurrent incidence of new onset diabetes, hypertension and hyperlipidemia. Furthermore, the question of whether the patients had diabetes was not verified in our laboratory. Finally, since the enzyme assays were run after a long storage time at –80^o^ C, we cannot be certain that there had not been a loss of activity.

In conclusion, we have found in a nested, case-control study that the GPx3 activity is negatively correlated with CVD mortality. This association appears to be limited to individuals with a low HDLc.
